# Supplementary material for: Container-based bioinformatics with Pachyderm
Source: Bioinformatics. 2018 Aug 8;35(5):839–46. doi: 10.1093/bioinformatics/bty699 (PMC6394392; doi:10.1093/bioinformatics/bty699)
Supplement: Supplementary Table 1 [file bty699_supplementary_table_1.pdf]

FeatureFinderMetabo

Number of workers	Job duration (s)	Job duration (h:m:s)	Serial processing time (s)	MEDIAN serial processing time (s)	Serial download time (s)	Serial upload time (s)	Speedup	Scaling efficiency
**20**	6561.00	01:49:21	114595.58	110829.78	178.26	365.54	**16.89**	**0.84**
**40**	3607.00	01:00:07	111655.97		196.45	299.78	**30.73**	**0.77**
**60**	2683.00	00:44:43	110003.58		248.94	300.92	**41.31**	**0.69**
**80**	2238.00	00:37:18	109495.48		357.37	369.00	**49.52**	**0.62**

findPeaks

Number of workers	Job duration (s)	Job duration (h:m:s)	Serial processing time (s)	MEDIAN serial processing time (s)	Serial download time (s)	Serial upload time (s)	Speedup	Scaling efficiency
**20**	2069.00	00:34:29	36898.79	37969.96	278.78	102.82	**18.35**	**0.92**
**40**	1208.00	00:20:08	38070.44		345.54	105.44	**31.43**	**0.79**
**60**	950.00	00:15:50	38133.36		606.19	109.88	**39.97**	**0.67**
**80**	688.00	00:11:28	37869.48		1227.99	129.60	**55.19**	**0.69**

CSI:FingerID

Number of workers	Job duration (s)	Job duration (h:m:s)	Serial processing time (s)	MEDIAN serial processing time (s)	Serial download time (s)	Serial upload time (s)	Speedup	Scaling efficiency
**20**	2036.00	00:33:56	38302.87	39283.41	260.64	1523.41	**18.65**	**0.93**
**40**	1055.00	00:17:35	38804.50		269.80	1636.71	**35.99**	**0.90**
**60**	788.00	00:13:08	39762.32		337.33	2173.80	**48.19**	**0.80**
**80**	604.00	00:10:04	42813.53		382.53	2619.93	**62.86**	**0.79**
